# Body-coil nonenhanced MR angiography using highly undersampled radial QISS

**DOI:** 10.1186/1532-429X-15-S1-O57

**Published:** 2013-01-30

**Authors:** Robert R Edelman, Shivraman Giri, Parag Amin, Ioannis Koktzoglou

**Affiliations:** 1 Radiology, NorthShore University HealthSystem, Evanston, IL, USA; 2 The University of Chicago Pritzker School of Medicine, Chicago, IL, USA; 3 Siemens Healthcare, Chicago, IL, USA; 4 Feinberg School of Medicine, Northwestern University, Chicago, IL, USA

## Background

Current approaches for magnetic resonance angiography (MRA) require the use of parallel imaging in order to keep scan times short and obtain adequate slice coverage and spatial resolution. However, parallel imaging necessitates the use of phased array coils, which increases setup time and restricts the vascular territory that can be imaged. We hypothesized that using Quiescent-Inflow Single-Shot (QISS) MRA with only the body coil for signal reception and a highly undesampled radial k-space trajectory could obviate the need for phased array coils.

## Methods

The Institutional Review Board approved the study. Two healthy subjects were imaged on a 1.5T MRI. In addition, four patients with peripheral arterial disease (PAD) were studied. The combination of contrast-enhanced TWIST and stepping table MRA was used as the reference standard. Body array, peripheral and spine phased array elements were used for signal reception with generalized auto-calibrating partially parallel acquisition (GRAPPA) and acceleration factor of 2 for CE-MRA and nonenhanced QISS MRA using a Cartesian k-space trajectory. For body-coil radial QISS, a matrix of 352 projections was obtained using either a single shot acquisition with 60 views and 2 signal averages (undersampling factor = 9.2) or a two-shot acquisition with 180 views and 1 signal average (undersampling factor = 6.1). A phase-based fat suppression technique was applied for two-shot, 180 view radial QISS only.

## Results

**Figure 1:**
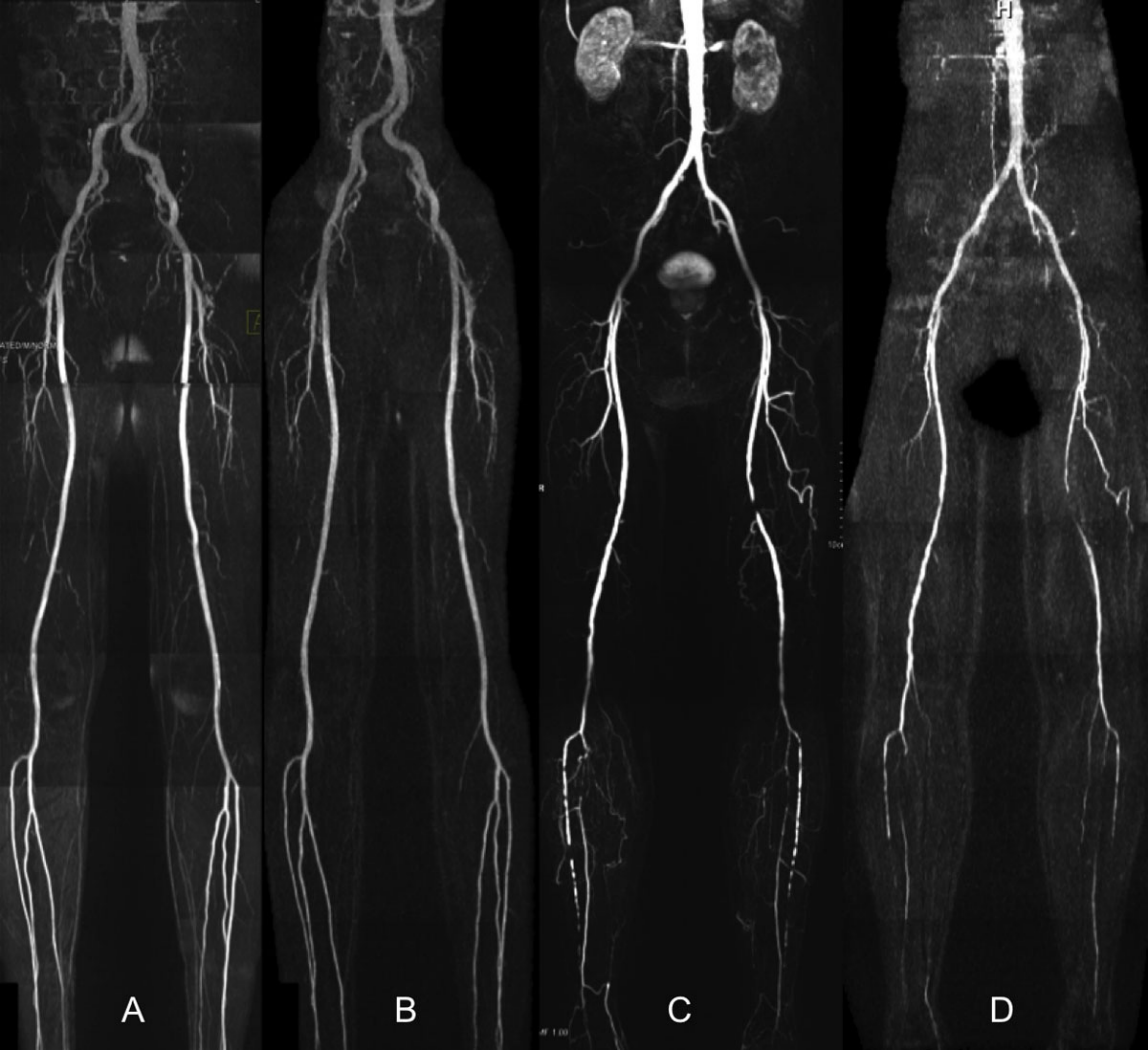
A) Cartesian QISS MRA in healthy subject obtained using phased array coils. B) Corresponding body-coil radial QISS shows more uniform fat suppression and vascular signal. C) CE-MRA in patient with PAD obtained using phased array coils. D) Corresponding body-coil radial QISS shows focal occlusion of left SFA and bilateral calf disease.

Scan time for body-coil radial QISS was approximately 13 minutes covering from the level of the ankles through the renal arteries. Diagnostic image quality for the peripheral arteries was obtained in all subjects. Vascular pathology in patients with PAD was well shown using body-coil radial QISS in comparison with CE-MRA using phased array coils (Figure 1). Compared with Cartesian QISS using phased array coils for signal reception, body-coil radial QISS showed more uniform fat suppression and vascular signal.

## Conclusions

Using body-coil radial QISS, diagnostic MR angiograms of the peripheral arteries can be obtained using acceleration factors up to 9.2. Streak artifacts from radial undersampling were minimized since QISS images are naturally sparse due to the combination of fat suppression and in-plane RF saturation. Moreover, by eliminating the need for phased array coils, this approach enables extended field of view vascular applications, including whole body vascular imaging, within practical scan times.

## Funding

This work was supported by The Grainger Foundation and 1R01HL096916.

